# 2470. A Survey of the Collection of Denominator Data in the Korean National Healthcare-associated Infections Surveillance System (KONIS), Intensive Care Unit Module

**DOI:** 10.1093/ofid/ofad500.2088

**Published:** 2023-11-27

**Authors:** Yee Gyung Kwak, Mi Suk Lee

**Affiliations:** Ilsan Paik Hospital, Ilsan, Kyonggi-do, Republic of Korea; Division of Infectious Diseases, Department of Internal Medicine, Kyung Hee University Hospital, Kyung Hee University School of Medicine, Seoul, Seoul-t'ukpyolsi, Republic of Korea

## Abstract

**Background:**

National surveillance data should be validated to identify data quality issues. While denominator data can have a significant effect on the healthcare-associated infection (HAI) rate, they are relatively overlooked compared to numerator data. The Korean National Healthcare-associated Infections Surveillance System (KONIS) has been conducting data validation, and this study analyzed the current status of, and problems with, collecting denominator data for the first time.

**Methods:**

The web-based cross-sectional survey was conducted on January 6, 2023 on 183 infection control nurses from 127 hospitals, among a total of 275 participating hospitals in the KONIS ICU module. The questionnaire was delivered using the QR code method and answered online by participants via SurveyMonkey^®^. The survey examined denominator data collection methods, such as patient days and device days of central lines and urinary catheters. The knowledge of the types of central lines and urinary catheters for KONIS reporting and the responses to case questions about specific situations related to the calculation of device days, were also evaluated.

**Results:**

The respective proportions of manual vs. electronic methods for counting denominator data were 10.4% vs. 81.4% for patient days, 22.4% vs. 67.8% for central line days, and 23.5% vs. 69.4% for urinary catheter days. About 27.3%, 29.5%, and 31.1% of respondents reported periodically checking the accuracy of patient days, central line days, and urinary catheter days data, respectively. Infection control nurses were responsible for collecting the majority (66.1%) of the denominator data and entering them into the Web-based Report and Analysis Program (88.0%). The survey results indicated that the rate of accurately understanding and responding to the case questions on device days for central lines ranged from 24.6% to 78.7%, while for urinary catheters, it ranged from 27.3% to 92.3%.

**Figure:**
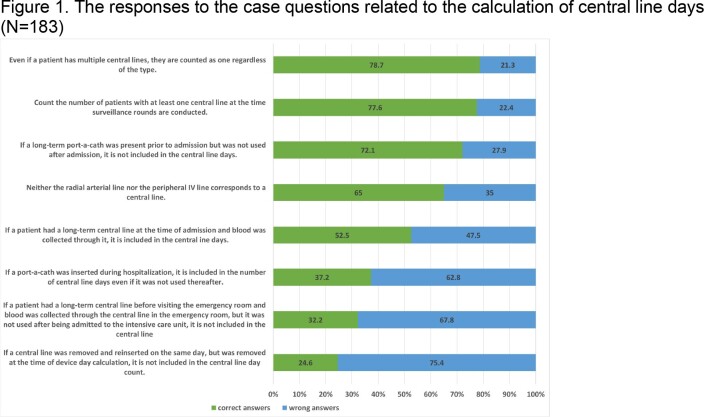

**Figure:**
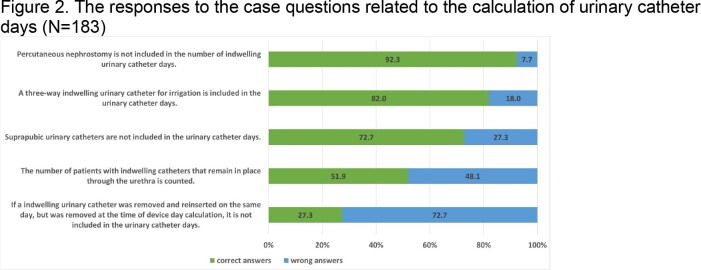

**Conclusion:**

To maintain the reliability of the national HAI surveillance data, it is important to collect accurate denominator data, in addition to numerator data. This study has identified several areas where improvements in denominator data collection methods are needed. Continuing education on the definition of denominator data and accurate data collection methods is crucial.

**Disclosures:**

**All Authors**: No reported disclosures

